# Individual and institutional predisposing factors of MRSA surgical site infection and outcomes—a retrospective case-control-study in 14 European high-volume surgical centres

**DOI:** 10.1093/jacamr/dlae046

**Published:** 2024-04-04

**Authors:** Jule Rutz, Jan-Hendrik Naendrup, Caroline Bruns, Annika Y Classen, Jon Salmanton-García, Harald Seifert, Rosanne Sprute, Jannik Stemler, Sarah V Walker, Oliver A Cornely, Blasius J Liss, Sibylle C Mellinghoff, SALT study group, Juliane Ankert, Louis Bernard, Camille Bataille, Elodie Couvé-Deacon, María Fernández Ferrer, Jesus Fortún, Alicia Galar, Thomas Guimard, Juan P Horcajada, Joan Mollar, Patricia Muñoz, Mathias W Pletz, Ferdinand Serracino-Inglott, Alex Soriano, Tim O Vilz

**Affiliations:** University of Cologne, Faculty of Medicine and University Hospital Cologne, Department I of Internal Medicine, Centre for Integrated Oncology Aachen Bonn Cologne Duesseldorf (CIO ABCD) and Excellence Centre for Medical Mycology (ECMM), Cologne, Germany; University of Cologne, Faculty of Medicine and University Hospital Cologne, Institute of Translational Research, Cologne Excellence Cluster on Cellular Stress Responses in Aging-Associated Diseases (CECAD), Kerpener Str. 62, 50937, Cologne, Germany; University of Cologne, Faculty of Medicine and University Hospital Cologne, Department I of Internal Medicine, Centre for Integrated Oncology Aachen Bonn Cologne Duesseldorf (CIO ABCD) and Excellence Centre for Medical Mycology (ECMM), Cologne, Germany; University of Cologne, Faculty of Medicine and University Hospital Cologne, Department I of Internal Medicine, Centre for Integrated Oncology Aachen Bonn Cologne Duesseldorf (CIO ABCD) and Excellence Centre for Medical Mycology (ECMM), Cologne, Germany; University of Cologne, Faculty of Medicine and University Hospital Cologne, Institute of Translational Research, Cologne Excellence Cluster on Cellular Stress Responses in Aging-Associated Diseases (CECAD), Kerpener Str. 62, 50937, Cologne, Germany; German Centre for Infection Research (DZIF), Partner Site Bonn Cologne, Cologne, Germany; University of Cologne, Faculty of Medicine and University Hospital Cologne, Department I of Internal Medicine, Centre for Integrated Oncology Aachen Bonn Cologne Duesseldorf (CIO ABCD) and Excellence Centre for Medical Mycology (ECMM), Cologne, Germany; University of Cologne, Faculty of Medicine and University Hospital Cologne, Institute of Translational Research, Cologne Excellence Cluster on Cellular Stress Responses in Aging-Associated Diseases (CECAD), Kerpener Str. 62, 50937, Cologne, Germany; German Centre for Infection Research (DZIF), Partner Site Bonn Cologne, Cologne, Germany; University of Cologne, Faculty of Medicine and University Hospital Cologne, Department I of Internal Medicine, Centre for Integrated Oncology Aachen Bonn Cologne Duesseldorf (CIO ABCD) and Excellence Centre for Medical Mycology (ECMM), Cologne, Germany; University of Cologne, Faculty of Medicine and University Hospital Cologne, Institute of Translational Research, Cologne Excellence Cluster on Cellular Stress Responses in Aging-Associated Diseases (CECAD), Kerpener Str. 62, 50937, Cologne, Germany; German Centre for Infection Research (DZIF), Partner Site Bonn Cologne, Cologne, Germany; German Centre for Infection Research (DZIF), Partner Site Bonn Cologne, Cologne, Germany; University of Cologne, Faculty of Medicine and University Hospital Cologne, Institute for Medical Microbiology, Immunology and Hygiene, Cologne, Germany; University of Cologne, Faculty of Medicine and University Hospital Cologne, Department I of Internal Medicine, Centre for Integrated Oncology Aachen Bonn Cologne Duesseldorf (CIO ABCD) and Excellence Centre for Medical Mycology (ECMM), Cologne, Germany; University of Cologne, Faculty of Medicine and University Hospital Cologne, Institute of Translational Research, Cologne Excellence Cluster on Cellular Stress Responses in Aging-Associated Diseases (CECAD), Kerpener Str. 62, 50937, Cologne, Germany; German Centre for Infection Research (DZIF), Partner Site Bonn Cologne, Cologne, Germany; University of Cologne, Faculty of Medicine and University Hospital Cologne, Department I of Internal Medicine, Centre for Integrated Oncology Aachen Bonn Cologne Duesseldorf (CIO ABCD) and Excellence Centre for Medical Mycology (ECMM), Cologne, Germany; University of Cologne, Faculty of Medicine and University Hospital Cologne, Institute of Translational Research, Cologne Excellence Cluster on Cellular Stress Responses in Aging-Associated Diseases (CECAD), Kerpener Str. 62, 50937, Cologne, Germany; German Centre for Infection Research (DZIF), Partner Site Bonn Cologne, Cologne, Germany; German Centre for Infection Research (DZIF), Partner Site Bonn Cologne, Cologne, Germany; University of Cologne, Faculty of Medicine and University Hospital Cologne, Institute for Medical Microbiology, Immunology and Hygiene, Cologne, Germany; University of Cologne, Faculty of Medicine and University Hospital Cologne, Department I of Internal Medicine, Centre for Integrated Oncology Aachen Bonn Cologne Duesseldorf (CIO ABCD) and Excellence Centre for Medical Mycology (ECMM), Cologne, Germany; University of Cologne, Faculty of Medicine and University Hospital Cologne, Institute of Translational Research, Cologne Excellence Cluster on Cellular Stress Responses in Aging-Associated Diseases (CECAD), Kerpener Str. 62, 50937, Cologne, Germany; German Centre for Infection Research (DZIF), Partner Site Bonn Cologne, Cologne, Germany; University of Cologne, Faculty of Medicine and University Hospital Cologne, Clinical Trials Centre Cologne (ZKS Köln), Cologne, Germany; HELIOS University Clinic of Wuppertal, Department of Haematology, Oncology, Palliative Care and Infectious Disease, Wuppertal, Germany; University of Witten, Faculty of Health, Witten, Germany; University of Cologne, Faculty of Medicine and University Hospital Cologne, Department I of Internal Medicine, Centre for Integrated Oncology Aachen Bonn Cologne Duesseldorf (CIO ABCD) and Excellence Centre for Medical Mycology (ECMM), Cologne, Germany; University of Cologne, Faculty of Medicine and University Hospital Cologne, Institute of Translational Research, Cologne Excellence Cluster on Cellular Stress Responses in Aging-Associated Diseases (CECAD), Kerpener Str. 62, 50937, Cologne, Germany; German Centre for Infection Research (DZIF), Partner Site Bonn Cologne, Cologne, Germany

## Abstract

**Objectives:**

To assess incidence rates of surgical site infections (SSI) by MRSA and to determine related factors and clinical outcome compared to MSSA, including country-specific, institutional and patient determinants.

**Patients and methods:**

We performed a subgroup analysis of the Europe-wide SALT (NCT03353532) study population with MRSA SSI from 14 centres in France, Germany, Italy, Spain and the UK.

**Results:**

An overall MRSA SSI incidence of 0.06% (*n* = 104) was found in 178 903 patients undergoing invasive surgery in 2016. Frequently observed comorbidities were chronic cardiovascular disease, diabetes and solid tumours. Compared to the overall MRSA SSI incidence, incidence rates were significantly higher in Spain (58 of 67 934 cases) and lower in Germany (16 of 46 443 cases; both *P *< 0.05). Centres with antibiotic stewardship (ABS) and infectious disease (ID) consultation programmes (*n* = 3/14) had lower MRSA rates (17 of 43 556 cases versus 61 of 83 048 cases, *P *< 0.05). In bivariate analyses, MRSA SSI patients were significantly older, had higher BMI and more comorbidities compared to MSSA (*P *< 0.05 each). Surgery performed between 6:00 and 12:00 pm led to higher MRSA proportions among *S. aureus* SSI (17 of 104 cases versus 62 of 640 cases, *P *< 0.05).

**Conclusions:**

This study shows low overall and country-specific incidence rates of MRSA SSI in Europe. We could show significant differences between countries as well as between centres with established ABS and ID consultation programmes were observed. The number of those programmes seems too small against this background.

## Introduction

Surgical site infections (SSI) are among the most frequent healthcare-associated infections (HAI).^1,2^ They are associated with prolonged hospitalization, poor clinical outcome and higher treatment costs.^3,4^ MSSA is the most common causative microorganism in the context of SSI.^2,5^ Despite its decreasing prevalence in Europe, MRSA continues to be of great importance due to increased morbidity, mortality and treatment costs in SSI patients compared to MSSA.^6^

The Europe-wide SALT (*Staphylococcus aureus* surgical site infection rates in five European countries) study recently analysed overall *S. aureus* SSI incidence in France, Germany, Italy, Spain and the UK for all invasive surgeries at 14 high-volume centres, defined as executing more than 10 000 procedures annually.^7^ Given the impact of MRSA on SSI outcomes, we report an in-depth subgroup analysis of patients with MRSA SSI within the SALT patient cohort. We evaluate patient and institutional determinants of MRSA incidence as well as overall and country-specific infection rates.

## Patients and methods

### Study design

This analysis was based on the dataset of the SALT study.^7,8^ SALT is a case-control study, comprising microbiologically proven SA SSI cases and matched uninfected controls without documented clinical or microbiological evidence of SSI (Table S2, available as Supplementary data at JAC-AMR Online) nested within a retrospective multinational, multicentre cohort of 178 903 patients undergoing invasive surgery at participating centres in France, Germany, Italy, Spain and the UK in 2016. Participating centres were Central University Hospital of Limoges, Central Regional University Hospital of Tours, Central Departmental Hospital of Vendée in France; University Hospitals of Munich, Bonn, Jena and Cologne in Germany; Hospital Clinic of Barcelona, General University Hospital Gregorio Marañón, Hospital del Mar Medical Research Institute, La Fe University and Polytechnic Hospital, University Hospital Ramón y Cajal Madrid in Spain; Central Manchester University Hospitals in UK and University of Udine and Santa Maria Misericordia University Hospital in Italy.

The primary objectives of the present analyses were to assess overall and country- and surgical speciality-specific incidence rates of MRSA SSI compared to MSSA SSI. Secondary objectives were to determine patient and institutional factors associated to MRSA SSI as well as to investigate the influence of MRSA SSI on clinical outcome compared to MSSA SSI. Exploratory objectives were the description of procedure-specific incidence of MRSA SSI and the impact of antibiotic stewardship (ABS) and ID consultation programmes on MRSA SSI incidence as well as their clinical outcome compared to MSSA SSI.

### Patient selection

From the SALT cohort, all 104 patients with MRSA SSI were included (Table S1). A total of 640 patients with MSSA SSI served as comparison group for bivariate analyses and, out of these, 104 cases served as controls for multivariate analyses. Twenty cases excluded from the SALT cohort due to missing matches were not included in our analysis.

### Data assessment

Regarding data capture we refer to the published report of the SALT main study.^8^ The following data were captured from all identified MRSA SSI patients as well as MSSA SSI patients: age, sex, BMI, American Society of Anaesthesiologists (ASA) score, comorbidities, length of hospitalization, required admission to ICU and length of ICU stay, reason for admission and attribution of admission to SSI (in case of ICU stay), necessity for surgical revision or re-admission, survival at day 30 and at day 90 after surgery, antibiotic treatment including its duration, patient functional status at admission and at final discharge, and death attributable to SSI as defined by the treating physician. If re-admission was necessary, reason for re-admission and attribution to SSI, duration of hospitalization and length of ICU stay as well as all antibiotic treatment and its duration were recorded. We further captured SA as causative pathogen from clinical specimens obtained during surgery or blood cultures including day of susceptibility testing, presence of resistance patterns (i.e. resistance to methicillin) and type of SSI according to ECDC criteria.^9^

Data on ABS programmes and infectious disease (ID) consultation were captured retrospectively by the American CDC Checklist for Core Elements of Hospital Antibiotic Stewardship^10^ and defined ‘established’ if introduced at least 1 year before January 2016.

### Statistical methods

MRSA SSI rates were determined for each country and each surgical specialty (e.g. vascular surgery) as well as individual surgical procedures (e.g. insertion of arteriovenous graft). For each incidence rate, the 95% confidence intervals for a binominal proportion were calculated. The cohort of MRSA SSI cases was compared to the cohort of MSSA SSI cases using both descriptive statistics and a logistic regression analysis. The dependent variable was infection by MRSA. Within the MRSA SSI cohort, we present continuous variables as mean (standard deviation) and median (interquartile range) and compared those using Mann–Whitney *U*-test after checking for normality using Kolmogorov–Smirnov test. Categorical variables are presented as proportions and compared using the Chi-square test or Fisher’s exact test. Cases with missing data were excluded from the respective calculations. To assess individual risk factors for MRSA SSI, a backward stepwise logistic regression analysis was performed. As a sensitivity analysis outcome measures were analysed in duplicate, once in the overall population and once in a subgroup of MRSA and MSSA patients matched one-to-one by a propensity score based on variables identified in the backward stepwise logistic regression analysis.

Statistical analysis and generation of all tables, listings and figures were performed using SPSS^®^ (IBM Statistics 27). Statistical significance was defined as *P* value less than 0.05. All tests were two-tailed.

### Ethics

The SALT study on which this retrospective study is based was submitted to the Research Ethics Commission of the University of Cologne (no. 17-078) for advice; the requirement for informed consent was waived due to the retrospective nature as well as the anonymous data capture strategy of this study. The study was registered at clinicaltrials.gov under NCT03353532.

## Results

In total, 764 of 178 903 [0.43% (95% CI, 0.40%–0.46%)] patients undergoing invasive surgery in 2016 in the 14 included European centres developed SA SSI; of these, 104 [14.0% (95% CI, 11.4%–16.3%)] infections were caused by MRSA accounting for an overall MRSA SSI incidence of 0.06% (95% CI, 0.05%–0.07%).

Table 1 shows the country-specific incidence rates. The single centre in Italy did not report MRSA cases. The highest country-specific MRSA SSI incidence rates were reported in Spain [0.09% (95% CI, 0.07%–0.11%), *n* = 58] and the UK [0.09% (95% CI, 0.04%–0.17%), *n* = 8]. The lowest rates were reported in Germany [0.03% (95% CI, 0.02%–0.06%), *n* = 16]. Compared among each other, these differences turn out to be significant in the case of Spain and Germany (both *P *< 0.05).

**Table 1.** Overall incidence of MRSA SSI per country

Centre	Number of included patients *N* = 178 903^a^	Number and percentage of MRSA SSI [*n* (%)] *N* = 104	Incidence rates of MRSA SSI [% (95% CI)]
France	35 974	22 (21.2)	0.06 (0.04–0.09)
Germany	46 443	16 (15.4)	0.03 (0.02–0.06)
Spain	67 934	58 (55.8)	0.09 (0.07–0.11)
UK	9168	8 (7.7)	0.09 (0.04–0.17)

Abbreviations: ID, infectious disease consultation service; Limoges, Central University Hospital of Limoges; Tours, Central Regional University Hospital of Tours; Vendée, Central Departmental Hospital of Vendée; LMU, University Hospital of Munich; UKB, University Hospital of Bonn; UKJ, University Hospital of Jena; UKK, University Hospital of Cologne; HCB, Hospital Clinic of Barcelona; HGGM, General University Hospital Gregorio Marañón; IMIM, Hospital del Mar Medical Research Institute; LaFe, La Fe University and Polytechnic Hospital; RyC, University Hospital Ramón y Cajal Madrid, NHS Manchester, Central Manchester University Hospitals NHS.

^a^Including the Italian centre.

Only three centres had established ABS programmes and ID consultation services, one in the UK and two in Germany (Table S4). Centres with ABS and ID consultancy programmes had both lower overall MRSA SSI incidences [0.04% (95% CI, 0.02%–0.06%) versus 0.07% (95% CI, 0.06%–0.09%), *P *< 0.05] and lower proportions of MRSA SSI among SA SSI [6.3% (95% CI, 4.0%–10.0%) versus 21.6% (95% CI, 17.3%–27.0%), *P *< 0.05]. Presence of ABS and ID consultation programmes did not significantly influence clinical outcomes. In these centres, re-admission rates and revision surgery rates were lower and 30 and 90 day survival were higher compared to centres without these services, whereas the necessity for ICU admission was higher (Tables S11 and S12).

**Figure 1.**
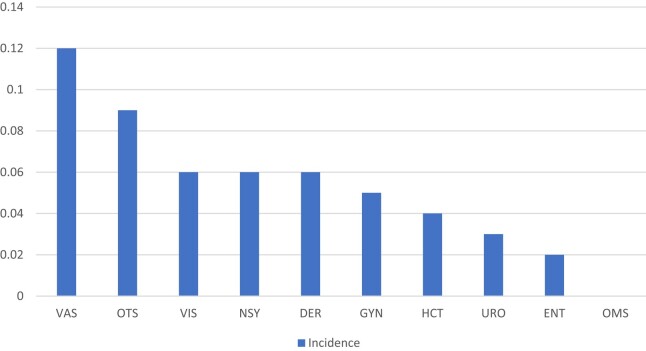
Incidence rates of MRSA SSI by category. Abbreviations: VAS, vascular surgery; OTS, orthopaedic and trauma surgery; VIS, visceral surgery; NSY, neurosurgery; DER, dermatological surgery; GYN, gynaecological surgery; HCT, heart and cardiothoracic surgery; URO, urological surgery; ENT, ear, nose and throat surgery; OMS, oral and maxillofacial surgery.

The highest specialty-specific incidence rates were observed in orthopaedic/trauma surgery [0.1% (95% CI 0.1%–0.1%), *n* = 31] and vascular surgery [0.1% (95% CI, 0.1%–0.2%), *n* = 15] (Table S5, Figure 1). In both specialties, patients were significantly more likely to develop MRSA SSI than patients in other specialties (both *P *< 0.05).

Regarding individual procedures, minimal invasive surgery of the bile duct had the highest procedure-specific incidence rate (2.3% [95% CI, 0.3%–15.8%], *n* = 1) (Table S5). In orthopaedic and trauma surgery, highest procedure-specific incidence rates were observed in revision surgeries, replacement and removal of prosthetic joint of the upper limb [0.5% (95% CI 0.1%–3.7%), *n* = 1], amputation and disarticulation of lower limb [0.3% (95% CI, 0.1%–0.8%), *n* = 4], spinal fusion [0.3% (95% CI, 0.1%–1.1%), *n* = 2] and primary total prosthetic replacement of hip joint [0.2% (95% CI, 0.1%–0.5%), *n* = 7]. The highest cumulative incidence of MRSA SSI was observed in primary total prosthetic replacement of hip joint and insertion of arteriovenous graft [both, 6.7% (95% CI, 3.3%–13.8%), *n* = 7].

Microbiological evidence was mostly obtained through wound swab (52.9%) or surgically obtained specimens (32.7%). Approximately 2% of infections were detected through blood culture (Table S3).

Patient characteristics are given in Table 2. Within the MRSA cohort, sex was evenly distributed (48.1% female, 51.9% male) and mean age was 65.9 (range, 21 to 95) years. Sixty (57.7%) of 104 MRSA SSI patients were overweight (BMI ≥25), 31 (29.8%) of these were obese (BMI ≥30). Most patients within the cohort had a mild or severe systemic disease (ASA 2–3). Frequently observed comorbidities were chronic cardiovascular disease (26.0%), diabetes (26.0%), solid organ malignancies (26.0%) and peripheral vascular disease (19.2%).

**Table 2.** Descriptive and bivariate analysis of demographics and patient characteristics

Characteristic	*S. aureus* SSI cases *N* = 744	MSSA SSI cases *N* = 640	MRSA SSI cases *N* = 104	Significance
Age [years]				
Mean (range)	58.1 (18–95)	56.8 (18–95)	65.9 (21–95)^a^	*P* < 0.05
Age groups [% (*n*)]				ns
18–29	9.5 (71)	10.6 (68)	2.9 (3)	
30–44	16.7 (124)	17.5 (112)	11.5 (12)	
45–59	20.4 (152)	20.6 (132)	19.2 (20)	
60–75	34.3 (255)	34.5 (221)	32.7 (34)	
>75	19.1 (142)	16.7 (107)	33.7 (35)	
Sex [% (*n*)]				ns
Female	48.1 (358)	48.1 (308)	48.1 (50)	
Male	51.9 (386)	51.9 (332)	51.9 (54)	
BMI [% (*n*)]^b^			^a^	*P* < 0.05
<18.5	2.1 (13)	2.2 (12)	1.2 (1)	
18.5–24.9	32.5 (203)	33.4 (181)	26.5 (22)	
25.0–29.9	34.4 (215)	34.3 (186)	34.9 (29)	
30.0–34.9	20.8 (130)	20.7 (112)	21.7 (18)	
35.0–39.9	6.9 (43)	6.1 (33)	12.0 (10)	
>40	3.4 (21)	3.3 (18)	3.6 (3)	
ASA [% (*n*)]^b^				ns
1	17.1 (101)	18.9 (94)	7.4 (7)	
2	41.2 (244)	39.8 (198)	48.9 (46)	
3	36.7 (217)	36.5 (182)	37.2 (35)	
4	4.9 (29)	4.6 (23)	6.4 (6)	
5	0.2 (1)	0.2 (1)	0.0 (0)	
ASA ≥ 2	82.9 (491)	81.1 (404)	92.6 (87)^a^	*P* < 0.05
Karnofsky performance status at admission [% (*n*)]^b^				ns
90%–100%	38.0 (220)	37.8 (186)	39.1 (34)	
70%–80%	40.6 (235)	40.0 (197)	43.7 (38)	
50%–60%	14.5 (84)	15.4 (76)	9.2 (8)	
30%–40%	5.5 (32)	5.1 (25)	8.0 (7)	
10%–20%	1.4 (8)	1.6 (8)	0.0 (0)	
Comorbidities [% (*n*)]				
Cardiovascular				
Chronic cardiovascular disease	23.1 (172)	22.7 (145)	26.0 (27)	
Congestive heart failure	7.7 (57)	7.5 (48)	8.7 (9)	
Peripheral vascular disease	12.1 (90)	10.9 (70)	19.2 (20)^a^	*P* < 0.05
Pulmonal				
Chronic obstructive pulmonary disease	6.2 (46)	5.3 (34)	11.5 (12)^a^	*P* < 0.05
Haematological/oncological				
Leukaemia	0.4 (3)	0.3 (2)	1.0 (1)	*P* = 0.147
Lymphoma	2.2 (16)	2.5 (16)	0.0 (0)	
Solid tumour	22.3 (166)	21.7 (139)	26.0 (27)	
Neurological				
Dementia	2.3 (17)	1.4 (9)	7.7 (8)^a^	*P* < 0.05
Transient ischaemic attack or cerebrovascular accident	5.8 (43)	4.8 (31)	11.5 (12)^a^	*P* < 0.05
Hemiplegia	1.3 (10)	1.3 (8)	1.9 (2)	
Other internal				
Diabetes	21.0 (156)	20.2 (129)	26.0 (27)	*P* = 0.177
Liver disease	4.4 (33)	3.9 (25)	7.7 (8)	*P* = 0.117
Chronic kidney disease	7.8 (58)	6.4 (41)	16.3 (17)^a^	*P* < 0.05
Other				
HIV/AIDS	1.2 (9)	1.4 (9)	0.0 (0)	
Procedure duration (min)				
Mean (range)	156.5 (0–1640)	154.2 (0–1640)	171.0 (15–725)	*P* = 0.083
Time of surgery [% (*n*)]				
0 to 6 am	2.6 (19)	2.7 (17)	1.9 (2)	
6 am to 12 am	52.9 (393)	54.0 (345)	46.2 (48)	
12 am to 6 pm	33.9 (252)	33.6 (215)	35.6 (37)	
6 pm to 12 pm	10.6 (79)	9.7 (62)	16.3 (17)^a^	*P* < 0.05

Abbreviations: MSSA, methicillin-susceptible *Staphylococcus aureus*; ns, not significant.

^a^Statistically significant difference between MRSA SSI cases and MSSA SSI cases.

^b^For BMI calculation, only 625 cases were included; for ASA score calculation, only 592 cases were included; for Karnofsky performance status, only 579 cases were included and for time of surgery, only 743 cases were included due to missing data in the remaining cases.

Bivariate analyses comparing MRSA SSI to MSSA cases showed that the MRSA SSI cohort was significantly older and had a higher BMI (both *P *< 0.05) (Table 2). Furthermore, MRSA SSI patients more frequently had at least mild systemic disease (ASA ≥2, *P *< 0.05) and were significantly more likely to have at least one comorbidity (*P *< 0.05), such as peripheral vascular disease, chronic obstructive pulmonary disease, dementia, transient ischaemic attack or cerebrovascular accident and chronic kidney disease (all *P *< 0.05). Surgery performed between 6:00 pm and 12:00 pm showed higher rates of MRSA compared to MSSA incidence rates (*P *< 0.05), whereas a statistically significant difference in procedure duration was not found (*P *= 0.083).

Risk factors for MRSA SSI compared to MSSA SSI are given in Table S6. We found age (OR 1.024; *P *< 0.001), surgery from 6 to 12 pm (OR 1.310; *P *= 0.070), chronic kidney disease (OR 2.148; *P *= 0.019) and dementia (OR 3.190; *P *= 0.029) to be predictive.

Regarding the outcome of MRSA SSI compared to MSSA (Table S7), mean length of hospitalization was significantly longer (24.9 versus 16.3 days; *P *< 0.05). Need for re-admission and for revision surgery were higher in MRSA SSI (58.7% versus 49.4% of cases and 57.7% versus 47.8% of cases) without reaching statistical significance (*P *= 0.079 and *P *= 0.062). MRSA SSI patients where numerically less likely to be admitted to the ICU (17.3% versus 19.7% of cases, *P *= 0.482). Overall survival after 30 and 90 days did not differ between MRSA and MSSA cases (Table S8). The observed differences did not prove to be significant after performing a propensity score matching (Tables S9 and S10), although hospitalization of patients with MRSA SSI continued to be longer than of patients with susceptible SA SSI (24.9 versus 19.7 days, *P *= 0.056).

## Discussion

This multicentre study shows a low overall and country-specific incidence of MRSA SSI in European high-volume surgical centres in line with the epidemiology of nosocomial MRSA infections in general.^11–14^ The overall MRSA rate of 14% is comparable to current European MRSA rates among invasive SA isolates as previously reported by ECDC.^11^ Our data show significant international differences.

The absolute numbers of MRSA SSI for each surgical procedure allow for exploratory analyses of procedures at risk for MRSA SSI and outcome-related assertions. In line with previous publications, MRSA SSI patients in our sample were older and had more underlying conditions than MSSA SSI patients.^15–17^

Procedure duration did not significantly affect the likelihood of MRSA SSI, whereas time of surgery affected MRSA SSI incidence rates. Patients undergoing surgery between 6:00 and 12:00 pm were more likely to develop MRSA SSI. On one hand, the time of the day and hours of work are known to affect behaviour and error rates of healthcare professionals.^18,19^ Lower adherence to hygiene and safety protocols might thus have contributed to the higher MRSA SSI incidence rates in patients who underwent surgery between 6:00 and 12:00 pm. However, several trials, including a cluster-randomized trial of 138 691 patients, have failed to demonstrate an effect of increased surgeon work hours on SSI rates or other clinically important outcomes.^20^ Patients operated during evening and night hours are structurally different from those during daytime and undergo different types of surgery (i.e. more acutely requiring surgery, fewer elective low-risk procedures). For the time-of-surgery subgroups, we saw no difference in mean age (67.5 versus 65.2 years), but more patients were above the age of 75 years (41.2% versus 32.2%) and most had higher baseline morbidity (ASA ≥3%–80% versus 35%) (Table S13). While our data implicate an influence of time point of surgery, many other factors such as colonization-status are of high relevance and must be investigated in forthcoming trials.

Moreover, our analysis shows longer hospitalization, higher rates of revision surgery and re-admission to hospital of patients hospitalized with MRSA SSI, not only causing higher costs but precipitating additional individual sequelae such as secondary HAI.^2,21^ MRSA SSI did not cause higher overall mortality.

Consistent with previous research,^22,23^ the presence of ABS programmes and ID consultation services was associated with significantly lower incidence rates of MRSA SSI compared to MSSA SSI in our cohort. The paucity of centres with established ID consult services (three out of 14) is concerning, considering their established efficacy and the focus of our trial on high-volume centres with research expertise on SSI. Greater efforts are required to further disseminate ID expertise considering the current inhomogeneity of training programmes as well as the distribution of ID specialists.^24^

Although being one of the largest MRSA SSI cohorts,^25,26^ limitations of our study are primarily the limited sample size resulting in more vulnerable findings. Our findings need to be interpreted with caution due to the limited number of centres included per country. Our findings may not match established distributions of MRSA in Europe. The single participating Italian centre, e.g. did not report any SSI due to MRSA in 2016, while the single UK centre had a particularly high incidence. In general, the SA SSI cohort established by the SALT study is more suitable for cross-national analyses rather than for regional epidemiological comparisons. The limited patient number is most salient in the lacking robustness of MRSA effects on outcomes. While the unmatched cohort showed a significant difference in duration of hospitalization, we were not able to reproduce this finding in the matched cohort, which did not meet the significance threshold (*P *= 0.056). Thus, conclusions regarding risk factors and outcomes drawn from our analyses may primarily add to the foundation of future studies.

This sub-cohort of the largest study of SSI in Europe confirms the low incidence of MRSA SSI, while highlighting its inhomogeneous extent even among leading surgical centres. As discussed, multiple factors influence the prevalence or MRSA SSI. Most concerning is the incomplete implementation of best practices approaches including ABS programmes and ID consultancy. Despite the inherent limitations, our findings support the urgent call for the establishment of these practices not only in high-volume surgical centres to further restrict the occurrence of multi-resistant pathogens.

## Supplementary Material

dlae046_Supplementary_Data

## Transparency declarations

Conflict of interest disclosure: All authors have completed the ICMJE uniform disclosure form at www.icmje.org/coi_disclosure.pdf and declare the following: BL received scientific grants from Pfizer. H.S. received grants or research support from the Bundesministerium für Bildung und Forschung (BMBF), Germany and Accelerate Diagnostics and has been a consultant for Debiopharm, Eumedica, Gilead, MSD and Shionogi. J.S.G. received speaker honoraria from Gilead and Pfizer outside the submitted work. J.S. received grants from the German Ministry of Education and Research and Basilea Pharmaceuticals, he has been a consultant for Produkt & Markt GmbH and Gilead, he received honoraria from Pfizer, AbbVie and Gilead. Moreover, he received support for attending meetings from the German Society for Infectious Diseases e.V. and the Meta-Alexander Foundation. He participates in a monitoring or advisory board of Micron Research and Alvea Vax. O.A.C. received grants from Amplyx, Basilea, BMBF, Cidara, EU-DG RTD (101037867), F2G, Gilead, MedPace, MSD, Mundipharma, Octapharma, Pfizer and Scynexis; he received personal fees for consulting, lectures, expert testimony or participation on monitoring and advisory boards from Abbott, Abbvie, Actelion, Al-Jazeera Pharmaceuticals, Allecra, Amplyx, Astellas, Biocon, Biosys, Cidara, Da Volterra, Entasis, Gilead, Grupo Biotoscana/United Medical/Knight, Hikma, IQVIA, Janssen, Matinas, MedPace, MedScape, MedUpdate, Menarini, Merck/MSD, Molecular Partners, MSG-ERC, Mylan, Noscendo, Noxxon, Octapharma, Paratek, Pardes, Pfizer, PSI, Pulmocide, Scynexis, Seres, Shionogi and The Prime Meridian Group. Beyond those, there is a patent planned (‘Geschlossene Inkubationssysteme mit verbessertem Atemwegszugang für Untersuchungsvorrichtungen’, DE 10 2021 113 007.7), filed by the University of Cologne and listing Oliver A. Cornely as one of three inventors. O.A.C. holds stocks from CoRe Consulting and EasyRadiology. He is Chair of Infectious Diseases Working Party (DGHO), Chair SWG Infections in Hematology (EHA), Advisory Committee Member (DGI), Educational Officer (ECMM), Treasurer (ISHAM), Board of Directors Member (MSG-ERC) and Editor-in-Chief for *Mycoses* (Wiley). R.S. received speaker honoraria from Pfizer. S.W. received consulting fees from the Institute for Quality and Efficiency in Health Care (IQWiQ). H.S., S.W., S.C.M. and O.A.C. received grants from the German Center for Infection Research. All other authors: none to declare.

## Access to data

All data in the study were made fully accessible for all authors. The corresponding author is responsible for the final submission for publication.

## Supplementary data

Tables S1–S13 are available as Supplementary data at *JAC-AMR* Online.
