# Supplementary material for: Individual and institutional predisposing factors of MRSA surgical site infection and outcomes—a retrospective case-control-study in 14 European high-volume surgical centres
Source: JAC Antimicrob Resist. 2024 Apr 4;6(2):dlae046. doi: 10.1093/jacamr/dlae046 (PMC10993902; doi:10.1093/jacamr/dlae046)
Supplement: dlae046_Supplementary_Data [file dlae046_supplementary_data.docx]

**Supplemental Table 1.** Inclusion and exclusion criteria

|  | Criteria |
| --- | --- |
| Inclusion | - Age 18 years or greater at the time of surgery |
| Exclusion | - Patients undergoing minimal invasive biopsies and eye surgery - SSI at the time of surgery - Cases with missing data defined as missing completely at random |

**Supplemental Table 2.** Study site data per country

| Characteristic | France | Germany | Italy | Spain | UK | Total |
| --- | --- | --- | --- | --- | --- | --- |
| Number of centres | 3 | 4 | 1 | 5 | 1 | 14 |
| Number of included patients | 35974 | 46443 | 19384 | 67934 | 9168 | 178,903 |

**Supplemental Table 3.** Microbiological data from *S. aureus* SSI cases

|  | *S. aureus* SSI [n (%)] N = 744 | MSSA SSI [n (%)] N = 640 | MRSA [n (%)] N = 104 |
| --- | --- | --- | --- |
| Type of SSI  Superficial incisional  Deep incisional  Organ space | 354 (46.3)  206 (27.0)  204 (26.7) | 307 (46.5)  172 (26.1)  181 (27.4) | 47 (45.2)  34 (32.7)  23 (22.1) |
| Specimen type  Aspirate  Blood culture  Sample obtained during surgery  Wound swab | 71 (9.5)  35 (4.7)  200 (26.9)  438 (58.9) | 58 (9.1)  33 (5.2)  166 (26.0)  383 (59.8) | 13 (12.5)  2 (1.9)  34 (32.7)  55 (52.9) |
| Wound class  Clean  Clean-contaminated  Contaminated  Dirty  Unknown | 373 (50.1)  154 (20.7)  98 (13.2)  61 (8.2)  58 (7.8) | 314 (49.1)  133 (20.8)  86 (13.4)  56 (8.8)  51 (8.0) | 59 (56.7)  21 (20.2)  12 (11.5)  5 (4.8)  7 (6.7) |

**Supplemental Table 4.** Overall incidence of MRSA SSI per centre and country

| Centre | Number of included patients N = 178903 | Number and percentage of MRSA SSI [n (%)] N = 104 | Incidence rates of MRSA SSI [% (95% CI)] N = 104 | ABS/ID on site |
| --- | --- | --- | --- | --- |
| France  Limoges  Tours  Vendée | 35974  9663  16697  9614 | 22 (21.2)  8 (7.7)  11 (10.6)  3 (2.9) | 0.06 (0.04 – 0.09)  0.08 (0.04 – 0.17)  0.07 (0.03 – 0.12)  0.03 (0.01 – 0.10) | unknown  unknown  no |
| Germany  LMU  UKB  UKJ  UKK | 46443  4946  7109  15581  18807 | 16 (15.4)  1 (1.0)  6 (5.8)  4 (3.8)  5 (4.8) | 0.03 (0.02 – 0.06)  0.02 (0.00 – 0.14)  0.08 (0.04 – 0.19)  0.03 (0.01 – 0.07)  0.03 (0.01 – 0.06) | no  no  yes  yes |
| Spain  HCB  HGGM  IMIM  LaFe  RyC | 67934  13041  15344  6555  12810  20184 | 58 (55.8)  7 (6.7)  21 (20.2)  7 (6.7)  12 (11.5)  11 (10.6) | 0.09 (0.07 – 0.11)  0.05 (0.03 – 0.11)  0.14 (0.09 – 0.20)  0.11 (0.05 – 0.22)  0.09 (0.05 – 0.16)  0.05 (0.03 – 0.10) | no  no  unknown  no  no |
| UK  NHS Manchester | 9168 | 8 (7.7) | 0.09 (0.04 – 0.17) | yes |
| Italy  Udine | 19384 | 0 (0.0) | 0.00 | no |

Abbreviations: ABS = antibiotic stewardship, ID = infectious disease consultation service, Limoges = Central University Hospital of Limoges, Tours = Central Regional University Hospital of Tours, Vendée = Central Departmental Hospital of Vendée, LMU = University Hospital of Munich, UKB = University Hospital of Bonn, UKJ = University Hospital of Jena, UKK = University Hospital of Cologne, HCB = Hospital Clinic of Barcelona, HGGM = General University Hospital Gregorio Marañón, IMIM = Hospital del Mar Medical Research Institute, LaFe = La Fe University and Polytechnic Hospital, RyC = University Hospital Ramón y Cajal Madrid, NHS Manchester = Central Manchester University Hospitals NHS, Udine = University of Udine and Santa Maria Misericordia University Hospital

**Supplemental Table 5.** Specialties and procedures with highest number and highest rate of MRSA SSI

| Procedure | Number and percentage of MRSA SSI [n (%)] N = 104 | Procedure specific incidence of  MRSA SSI [% (95% CI)] N = 104 |
| --- | --- | --- |
| Dermatological surgery  Wound debridement  Incision and excision of skin and subcutaneous tissue  Skin autograft transplantation to head or neck  Facial plastic surgery | 10 (9.6)  5 (4.8)  3 (2.9)  1 (1.0)  1 (1.0) | 0.1 (0.0 – 0.1)  0.1 (0.1 – 0.3)  0.0 (0.0 – 0.1)  0.1 (0.0 – 0.8)  0.1 (0.0 – 0.7) |
| Ear, nose and throat surgery | 2 (1.9) | 0.0 (0.0 – 0.1) |
| Gynaecological surgery  Caesarean section  Breast excision and resection  Surgery on vagina and recto-uterine pouch  Plastic surgery of breast augmentation and reduction  Surgery on uterus and cervix uteri | 12 (11.5)  6 (5.8)  3 (2.9)  1 (1.0)  1 (1.0)  1 (1.0) | 0.1 (0.0 – 0.1)  0.1 (0.0 – 0.2)  0.1 (0.0 – 0.3)  0.1 (0.0 – 0.7)  0.1 (0.0 – 0.7)  0.0 (0.0 – 0.2) |
| Heart and cardiothoracic surgery  Revascularization of the heart  Open surgery of the lung and pleura (reconstruction and removal)  Atrial septum/valve repair surgery | 4 (3.9)  2 (1.9)  1 (1.0)  1 (1.0) | 0.0 (0.0 – 0.1)  0.1 (0.0 – 0.4)  0.1 (0.0 – 0.4)  0.0 (0.0 – 0.3) |
| Neurosurgery  Operations on scull, brain, meninges  Reconstruction of a nerve and nerve plexus  Access to the vertebral column, extradural  Operations on spinal cord and spinal cord structures | 7 (6.7)  3 (2.9)  2 (1.9)  1 (1.0)  1 (1.0) | 0.1 (0.0 – 0.1)  0.1 (0.0 – 0.2)  0.1 (0.0 – 0.2)  0.1 (0.0 – 1.0)  0.1 (0.0 – 0.6) |
| Oral and maxillofacial surgery | 0 (0.0) | 0.0 |
| Orthopaedic and trauma surgery  Primary total prosthetic replacement of hip joint  Amputation and disarticulation of lower limb  Open repair of a fractured long tubular bone  Operations on bone  Operations on the vertebral column  Spondylodesis  Open reduction and internal fixation of a fracture or slipped epiphysis  Revision, replacement and removal of prosthetic joint of the upper limb  Reduction of a joint dislocation | 31 (29.8)  7 (6.7)  4 (3.9)  4 (3.9)  4 (3.9)  3 (2.9)  2 (1.9)  2 (1.9)  1 (1.0)  1 (1.0) | 0.1 (0.1 – 0.1)  0.2 (0.1 – 0.5)  0.3 (0.1 – 0.8)  0.1 (0.0 – 0.3)  0.1 (0.0 – 0.2)  0.1 (0.0 – 0.2)  0.3 (0.1 – 1.1)  0.1 (0.0 – 0.3)  0.5 (0.1 – 3.7)  0.3 (0.0 – 1.7) |
| Urological surgery  Open surgery on the urethra  Open surgery on the prostate  Transplantation of kidney  Minimally invasive operations on the prostate  Minimally invasive operations on the bladder | 5 (4.8)  1 (1.0)  1 (1.0)  1 (1.0)  1 (1.0)  1 (1.0) | 0.0 (0.0 – 0.1)  0.3 (0.0 – 2.0)  0.2 (0.0 – 1.3)  0.1 (0.0 – 0.6)  0.1 (0.0 – 0.4)  0.0 (0.0 – 0.2) |
| Vascular surgery  Insertion of arteriovenous graft  Ligation of blood vessels  Angioplasty  Surgery on lymph nodes and vessels  Open insertion of stent grafts | 15 (14.4)  7 (6.7)  2 (1.9)  2 (1.9)  2 (1.9)  1 (1.0) | 0.1 (0.1 – 0.2)  0.4 (0.2 – 0.8)  0.7 (0.2 – 2.9)  0.6 (0.2 – 2.3)  0.1 (0.0 – 0.3)  0.2 (0.0 – 1.3) |
| Visceral surgery  Open resection of large intestine  Operations on the abdominal wall  Open excision and resection of pancreas  Minimally invasive resection of large intestine  Minimally invasive surgery of the bile duct | 18 (17.3)  3 (2.9)  3 (2.9)  2 (1.9)  2 (1.9)  1 (1.0) | 0.1 (0.0 – 0.1)  0.2 (0.1 – 0.6)  0.1 (0.0 – 0.3)  0.3 (0.1 – 1.3)  0.2 (0.1 – 0.8)  2.3 (0.3 – 15.8) |

**Supplemental Table 6** Multivariate analysis of significant differences between MSSA and MRSA SSI cases (Logistic regression)

| Factor | Significance | Odds ratio | |
| --- | --- | --- | --- |
| Age | p=0.000 | 1.024 | |
| Surgery 6 pm to 12 pm | p=0.070 | 1.310 | |
| CKD | p=0.019 | 2.148 | |
| Dementia | p=0.029 | 3.190 | |
| Included variables | | |  |
| - Surgical category - Age - Procedure duration - Diabetes - Sex - Smoking status - Time of surgery - Liver disease - Solid tumour - HIV/AIDS - Chronic kidney disease (CKD) - Chronic heart failure (CHF) - Chronic cardiovascular disease (CVD) - Chronic obstructive pulmonary disease (COPD) - Peripheral vascular disease (PVD) - Cerebrovascular accident/transient ischemic accident (CVA/TIA) - Dementia - Hemiplegia - Connective tissue disease (CTD) - Leukaemia - Malignant lymphoma - Peptic ulcer | | |  |

**Supplemental Table 7.** Complications compared between MRSA and MSSA SSI cases

| Complication | MRSA N = 104 | MSSA N = 640 | Significance |
| --- | --- | --- | --- |
| Mean hospitalization [days (range)] | 24.89 (0 – 133) | 16.26 (0 – 180) | p<0.05 |
| ICU stay following surgery [% (n)] | 17.3 (18) | 19.7 (126) | p=0.569 |
| Readmission to hospital [% (n)] | 58.7 (61) | 49.4 (316) | p=0.079 |
| Revision surgery [% (n)] | 57.7 (60) | 47.8 (306) | p=0.062 |

**Supplemental Table 8.** Survival compared between MRSA and MSSA cases

| Survival | MRSA N = 104 | MSSA N = 640 | Significance |
| --- | --- | --- | --- |
| 30 days [% (n)] | 96.2 (100) | 97.3 (623) | p=0.519 |
| 90 days [% (n)] | 89.4 (93) | 91.9 (588) | p=0.405 |

**Supplemental Table 9.** Complications compared between MRSA and MSSA cases with propensity score matching

| Complication | MRSA N = 104 | MSSA N = 104 | Significance |
| --- | --- | --- | --- |
| Mean hospitalization [days (range)] | 24.89 (0 – 133) | 19.66 (0 – 180) | p=0.056 |
| ICU stay following surgery [% (n)] | 17.3 (18) | 21.2 (22) | p=0.482 |
| Readmission to hospital [% (n)] | 58.7 (61) | 50.0 (52) | p=0.210 |
| Revision surgery [% (n)] | 57.7 (60) | 48.1 (50) | p=0.165 |

**Supplemental Table 10.** Survival compared between MRSA and MSSA cases with propensity score matching

| Survival | MRSA N = 104 | MSSA N = 104 | Significance |
| --- | --- | --- | --- |
| 30 days [% (n)] | 96.2 (100) | 95.2 (99) | p=0.522 |
| 90 days [% (n)] | 89.4 (93) | 86.5 (90) | p=1.000 |

**Supplemental Table 11.** Complications of MRSA SSI compared between centres with and without established ABS and ID consultation programmes

| Complication | ABS/ID on site N = 17 | No ABS/ID on site N = 61 | Significance |
| --- | --- | --- | --- |
| Mean hospitalization [days (range)] | 28.18 (0 – 133) | 26.05 (1 – 94) | p=0.569 |
| ICU stay following surgery [% (n)] | 23.5 (4) | 19.7 (12) | p=0.740 |
| Readmission to hospital [% (n)] | 41.2 (7) | 57.4 (35) | p=0.236 |
| Revision surgery [% (n)] | 41.2 (7) | 55.7 (34) | p=0.288 |

**Supplemental Table 12.** Survival of MRSA SSI compared between centres with and without established ABS and ID consultation programmes

| Survival | ABS/ID on site N = 17 | No ABS/ID on site N = 61 | Significance |
| --- | --- | --- | --- |
| 30 days [% (n)] | 100 (17) | 95.1 (58) | p=0.591 |
| 90 days [% (n)] | 100 (17) | 88.5 (54) | p=0.202 |

**Supplemental Table 13.** Demographics and characteristics of patients with MRSA surgical site infections (SSI) undergoing surgery between 6 pm and 12 pm compared to other daytimes

| Characteristic | Time of surgery 6 pm to 12 pm N = 17 | All other daytimes N = 87 |
| --- | --- | --- |
| Age [years]  Mean (range)  Age groups [% (n)]  18 – 29  30 – 44  45 – 59  60 – 75  >75 | 67.5 (27 – 95)  5.9 (1)  11.8 (2)  17.6 (3)  23.5 (4)  41.2 (7) | 65.2 (21 – 93)  3.4 (3)  10.3 (9)  20.7 (18)  33.3 (29)  32.2 (28) |
| Sex [% (n)]  Female  Male | 35.3 (6)  64.7 (11) | 50.6 (44)  49.4 (43) |
| BMI [% (n)] *  <18.5  18.5 – 24.9  25.0 – 29.9  30.0 – 34.9  35.0 – 39.9  >40 | 10.0 (1)  40.0 (4)  20.0 (2)  10.0 (1)  20.0 (2)  0.0 (0) | 0.0 (0)  24.6 (15)  32.8 (20)  26.2 (16)  11.5 (7)  4.9 (3) |
| ASA [% (n)] *  1  2  3  4  5 | 0.0 (0)  18.8 (3)  68.8 (11)  12.5 (2)  0.0 (0) | 9.0 (7)  55.1 (43)  30.8 (24)  5.1 (4)  0.0 (0) |
| Karnofsky performance status at Admission [% (n)] *  90 – 100%  70 – 80%  50 – 60%  30 – 40%  10 – 20% | 40.0 (6)  46.7 (7)  0.0 (0)  13.3 (2)  0.0 (0) | 38.9 (28)  43.1 (31)  11.1 (8)  6.9 (5)  0.0 (0) |

* For BMI calculation, only 71 cases were included; for ASA score calculation, only 94 cases were included and for Karnofsky performance status, only 87 cases were included due to missing data in the remaining cases.
